# StratGAN: Conditional Adversarial Network for Permittivity Inversion of Borehole Radar Data in Stratified Media

**DOI:** 10.3390/s26102946

**Published:** 2026-05-08

**Authors:** Song Qing, Ding Yang, Raffaele Persico, Cheng Guo, Chuanhao Hu, Jianjian Huo, Jisheng Tong, Jinsong Liang, Qing Zhao

**Affiliations:** 1 College of Nuclear Technology and Automation Engineering, Chengdu University of Technology, Chengdu 610059, China; qingsong@stu.cdut.edu.cn (S.Q.); huchuanhao17@cdut.edu.cn (C.H.); 2 School of Resources and Environment, University of Electronic Science and Technology of China, Chengdu 611731, China; guocheng@uestc.edu.cn (C.G.); zhaoq@uestc.edu.cn (Q.Z.); 3 Department of Engineering of the Environment and the Territory DIAM, University of Calabria, Via P. Bucci, Cubo 45 A, 87036 Rende, Italy; raffaele.persico@unical.it; 4 Faculty of Electrical Engineering and Computer Science, Ningbo University, Ningbo 315211, China; huojianjian@nbu.edu.cn; 5 Guangxi Key Laboratory of Multimedia Communications and Network Technology, School of Computer, Electronics and Information, Guangxi University, Nanning 530004, China; jisheng.tong@gxu.edu.cn; 6 Northeast Institute of Geography and Agroecology, Chinese Academy of Sciences, Harbin 150081, China; liangjinsong@iga.ac.cn

**Keywords:** borehole radar (BHR), stratified media, adversarial neural network, deep neural networks, StratGAN

## Abstract

An ill-posed permittivity inversion problem is encountered in borehole radar (BHR) applications within stratified media due to a highly nonlinear forward relation, insufficient statistical coverage under data-limited conditions, strong noise contamination, and limited borehole observation geometry, which together cause instability and blurred boundaries. To address these challenges, a stratified media oriented conditional generative adversarial network for permittivity inversion, termed StratGAN, is proposed. BHR waveform data are used as the conditional input, and the complex mapping from time domain waveforms to depth domain permittivity distributions is learned end to end through conditional adversarial training between a generator and a discriminator, jointly constrained by a composite loss. During training, statistical characteristics of layered structures are learned from real samples by the discriminator, and adaptive feedback is provided as a data-driven loss to suppress spurious structures and boundary ambiguity. WGAN-GP is adopted and combined with a patch-based local discrimination mechanism to reinforce high-frequency details and geometric boundary consistency, thereby reducing the over-smoothing tendency of conventional CNNs. In addition, geometric consistency of inversion results is improved in an end-to-end manner without relying on complicated velocity analysis. Quantitative evaluations on simulated and measured datasets indicate that, compared with an architecture-matched convolutional neural network (CNN) and the baseline model GPRNet, StratGAN achieves overall better performance in terms of mean absolute error, coefficient of determination, and structural similarity metrics, and layered interfaces and anomaly boundaries are more effectively recovered. For the controlled measured data, the coefficient of determination ($R^{2}$) is improved to 0.9533 by StratGAN, whereas a value of 0.5598 is obtained by GPRNet. These results indicate the potential of StratGAN to enhance the reliability and structural fidelity of BHR permittivity inversion under limited-sample conditions, and preliminary evidence is provided for its practical applicability under controlled measured conditions.

## 1. Introduction

With the continued advancement of subsurface resource development and underground space utilization, the demand for fine scale imaging and quantitative characterization of deep geological bodies is continuously increased [1,2,3]. Borehole radar (BHR) is implemented by deploying transmitting and receiving antennas inside a borehole, radiating high-frequency pulsed electromagnetic waves into the surrounding medium, and recording scattered echoes, thereby enabling high-resolution sensing of the near borehole region [4,5]. Compared with surface ground penetrating radar (Ground Penetrating Radar, GPR), the near target observation geometry of BHR is associated with a shorter propagation path, and the absorption and attenuation of high-frequency electromagnetic waves by highly conductive near surface cover, such as clays and aquifers, are partially reduced. As a result, the signal-to-noise ratio and geometric fidelity for deep target detection are improved [6,7]. In recent years, borehole radar has been applied to hydrocarbon reservoir characterization [8], ore body boundary delineation [9], karst fracture detection, site selection for nuclear waste repositories, and groundwater contamination monitoring. Its meter scale investigation range and centimeter scale resolution are positioned between point scale well logging measurements and areal seismic measurements, and an important complement is provided in this intermediate scale [10,11,12,13].

BHR is characterized by high sensitivity to permittivity perturbations, and the amplitude and phase responses of echoes are linked to lithology and pore fluid information, which provides a physical basis for quantitative inference from waveforms to lithology and fluids. Permittivity is often governed by pore fluid type and saturation, and thus water bearing and hydrocarbon bearing media and their interfaces are typically associated with differentiated electromagnetic responses. This mechanism is also used for quantitative characterization of hydrological parameters such as water content [14]. However, stronger ill posedness is introduced into BHR imaging and permittivity inversion by the pervasive presence of layered media. In sedimentary hydrocarbon reservoirs, pronounced vertical heterogeneity and abrupt contrasts are formed by interbedding among sandstone reservoirs, mudstone or shale caprocks, and surrounding rocks [15]. In volcanic reservoirs, lithological and petrophysical differences are further intensified by composite sequences formed by multi-stage lava flows and pyroclastic rocks [16]. Velocity stratification and interlayer refraction induced by layered structures are associated with enhanced travel time nonlinearity, including non-hyperbolic moveout, wavefront bending, and multipath propagation. Consequently, imaging focus and positioning accuracy are degraded for conventional methods built on constant velocity or smoothly varying velocity assumptions, and the non-uniqueness and noise sensitivity of permittivity inversion are worsened [17,18,19]. Therefore, under layered media conditions, stable recovery of permittivity parameters for layer boundaries and anomalous bodies under limited observations and noise interference is identified as a critical bottleneck for quantitative BHR applications.

Within the model-driven paradigm, Kirchhoff and FK migration are commonly used for rapid field interpretation because relatively high computational efficiency and clear physical meaning are offered [20,21]. Reverse time migration (RTM) and full waveform inversion (FWI) are formulated through wave equation solving and joint matching of amplitude and phase, and higher-resolution parameter reconstruction is expected in principle. However, strong sensitivity to the velocity model, the initial model, and numerical stability is often exhibited by these methods. In layered media, refraction and non-hyperbolic moveout are responsible for systematic geometric errors introduced during migration and time-to-depth conversion. Under the high-frequency radar regime, the objective function is made more prone to multimodality and cycle skipping by the lack of low-frequency content, while substantial computational and memory costs are incurred, which limits engineering practicality [22,23,24]. Therefore, in strongly ill-posed BHR scenarios for layered media, more robust prior constraints and stabilization mechanisms are still required and are of practical significance [10,25].

Deep learning is increasingly advanced for tasks such as anomaly detection and feature recognition in GPR, whereas research on quantitatively inverting permittivity distributions directly from radar data remains relatively limited. Alvarez et al. formulate this problem as an image-to-image mapping task, and reconstructions of regular subsurface defects are achieved using encoder decoder networks, UNet, and generative adversarial networks [26]. The feasibility of recovering structural representations from radar data is demonstrated, but quantitative reconstruction of permittivity is not further realized. Ji et al. propose a DNN-based inversion network, in which a mapping from GPR data to permittivity images is established via temporal dimension compression and global feature encoding [27]. Leong and Zhu [28] propose GPRNet, a CNN-based framework in which electromagnetic velocity is directly inverted from zero offset GPR traces. The model is trained on large-scale synthetic trace velocity pairs and is validated on both synthetic and field data, with results being consistent with prior inversion models. Liu et al. propose GPRInvNet, where relative permittivity is estimated through the fusion of adjacent trace features and feature compression [29]. Xie et al. propose an improved UNet encoder–decoder framework that consists of a data compression module, a UNet, and an output module, and permittivity inversion is implemented [30]. Dai et al. propose 3DInvNet, where attention-based prior denoising is integrated with a 3D UNet with multiscale feature aggregation for three-dimensional permittivity inversion from GPR C scan data. Nonlinearity, ill posedness, and high computational cost in conventional iterative methods are alleviated, and robustness and generalization are validated on numerical and measured data [31]. Esposito et al. introduce an optimized UNet variant into radar inversion tasks, and the feasibility of mapping from the data space to the electromagnetic parameter space by deep networks is further verified [32]. Overall, neural network-based methods are typically characterized by fast inference and weaker dependence on initial models, but performance is still tightly coupled to the representativeness and scale of the training data, and stability and generalization under limited sample conditions are still required to be strengthened with task-specific mechanisms. In addition, it should be emphasized that, in recent years, deep learning-based radar inversion methods, including GPRInvNet, improved UNet variants, and 3DInvNet, have achieved important progress under their respective task settings, data organization schemes, and acquisition configurations. However, these methods cannot always be compared directly in a strict sense, because some models are designed for two-dimensional Bscan inversion, some rely on adjacent trace feature fusion, and some are specifically developed for three-dimensional C-scan volumetric inversion. Therefore, numerical superiority or inferiority reported across different studies should be interpreted with caution unless reimplementation and retraining are conducted under a unified dataset, a unified training protocol, and a unified evaluation framework.

It is worth noting that, when CNN or UNet models driven primarily by pointwise regression losses are directly applied to data-limited BHR inversion in layered media, three mechanism related issues are likely to be encountered. First, details and boundaries are prone to being averaged out. Pointwise losses such as MSE and MAE tend to fit the central tendency of the conditional distribution. When strong non-uniqueness or multimodality is present, texture smoothing and boundary blurring are induced, and layer interfaces and small-scale anomaly edges are rendered less sharp [33]. Second, a tradeoff is imposed between small target representation and global fitting. Anomalies such as fractures and voids usually occupy a small area, whereas the layered background dominates. When the loss function is more sensitive to global pixel averages, background errors are preferentially reduced, but insufficient local contrast or morphological bias in anomalies is produced [34]. Third, distribution bias is made more pronounced under limited samples. High-quality labels are often derived from cores, well logs, or controlled experiments, but sample construction is costly. When training samples are scarce and noise conditions vary, the network is more easily affected by data distribution shift, and performance across borehole intervals or operating conditions is prone to fluctuations [35].

Against this backdrop, conditional generative adversarial networks (CGANs) are regarded as a viable route for improving structural consistency and detail recovery. Compared with DNNs that rely only on pointwise regression losses, CGANs emphasize output distribution matching and structural detail characterization through adversarial learning, while conditional constraints are also used to enhance the stability of generated results. CGANs are validated in a broad range of image-to-image tasks, and potential advantages over conventional encoder–decoder frameworks are exhibited in certain scenarios. To address potential instability in adversarial training, improved objectives and regularization terms are commonly introduced to enhance training controllability [33]. Meanwhile, electromagnetic inversion is viewed as a mapping from the observation domain to the parameter domain, and modeling similarities to the image translation paradigm are shared [33,36].

To cope with the strong ill posedness and data-limited constraints in layered media BHR permittivity inversion, StratGAN is proposed in this work. First, conditional adversarial training between a generator and a discriminator is performed, the statistical characteristics of layered structures are learned by the discriminator from real samples, and an adaptive loss function is provided through feedback to mitigate spurious structures and boundary ambiguity. Second, WGAN-GP is adopted and is combined with a patch-based local discrimination mechanism, by which local high-frequency details and boundary consistency are reinforced and excessive smoothing is reduced. Third, the correspondence between time-domain waveforms and depth domain permittivity parameters is learned in an end-to-end manner, and geometric consistency in inversion is improved without explicit reliance on complex velocity analysis. Overall, an implementable scheme that integrates adversarial priors with end-to-end mapping is provided by StratGAN for quantitative permittivity inversion of layered media BHR, which is expected to enhance the reliability and usability of borehole radar inversion results.

## 2. Methodology

### 2.1. Characteristics of the BHR Inversion Task

The forward modeling process of borehole radar can be abstracted as a complex nonlinear operator, in which the radar observation response is computed from the spatial distribution of electromagnetic parameters in the formation surrounding the borehole. By moving the borehole radar, the received waveform $d_{r}\left(t\right)$ is recorded. Let F denote the mapping between the permittivity distribution $\varepsilon_{r}$ in the investigation region and the sampled and discretized electric field values *d* derived from $d_{r}\left(t\right)$, which is expressed as follows:

(1) $$d=F\;\left(\varepsilon_{r}\right)$$

During inversion, the permittivity distribution in the investigation region is solved from the measured electric field values, which is written as:

(2) $$\varepsilon_{r}=F^{-1}\;\left(d\right)$$

Accordingly, the inversion task is formulated as recovering the permittivity map of the investigation region that corresponds to the acquired electric field values. The core of deep learning-based inversion is to approximate the highly complex inverse operator $F^{-1}$ by exploiting the strong nonlinear representation capability of neural networks. To improve clarity of presentation, the main notations used in this study are unified as follows. The continuously received radar waveform is denoted by $d_{r}\left(t\right)$, and the sampled and discretized data used as the network input are denoted by *d*. The relative permittivity distribution within the investigation region is denoted by $\varepsilon_{r}$. The ground-truth reference permittivity model is denoted by $\varepsilon_{r}^{\mathrm{ref}}$. The permittivity model generated by the generator is denoted by $\varepsilon_{r}^{G}=G\left(d\right)$. The interpolated sample used in the gradient penalty term is denoted by $\varepsilon_{r}^{I}$. In addition, *G* and *D* denote the generator and the discriminator, respectively.

### 2.2. Architecture of StratGAN

The architecture of StratGAN consists of a generator and a discriminator, both of which are designed by fully considering the characteristics of the borehole radar inversion task and the requirements of GAN training. The generator in StratGAN adopts a classical encoder–decoder structure. Its primary function is to compress the input Bscan image of borehole radar into a low-dimensional latent feature space that contains essential information, and then to progressively decompress and refine these features through the decoder, so that a high-resolution permittivity distribution map is finally reconstructed.

**Figure 1:**
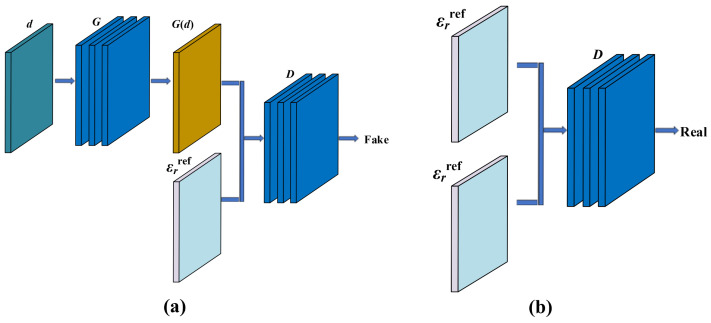
Training of StratGAN, where the discriminator *D* learns to distinguish fake and real data pairs. (**a**) Framework in which *D* learns to distinguish fake pairs. Here, *G* denotes the generator, *D* denotes the discriminator, $\varepsilon_{r}^{\mathrm{ref}}$ denotes the real data, and G(d) denotes the data generated by *G* conditioned on *d*. The discriminator *D* is trained to classify the pair $\left(\varepsilon_{r}^{\mathrm{ref}},G\left(d\right)\right)$. (**b**) Framework in which *D* learns to distinguish real pairs. The discriminator *D* is trained to classify the pair $\left(\varepsilon_{r}^{\mathrm{ref}},\varepsilon_{r}^{\mathrm{ref}}\right)$.

The discriminator *D* used in StratGAN training is trained to distinguish fake data, and an overview of the corresponding framework is shown in Figure 1a. Here, the generator is denoted by *G*, and the discriminator is denoted by *D*. The real data are denoted by $\varepsilon_{r}^{\mathrm{ref}}$, and G(d) denotes the output generated by *G* conditioned on the input data *d*. The discriminator network *D* takes two inputs, namely the pair $\left(G\left(d\right),\varepsilon_{r}^{\mathrm{ref}}\right)$. In contrast, the framework in which the discriminator is trained to distinguish real data is summarized in Figure 1b. In this case, the discriminator network *D* also takes two inputs, both of which are $\varepsilon_{r}^{\mathrm{ref}}$, that is, the pair $\left(\varepsilon_{r}^{\mathrm{ref}},\varepsilon_{r}^{\mathrm{ref}}\right)$. After extensive training, the generator *G* is expected to produce images that cannot be distinguished by the discriminator *D*. Conversely, after extensive training, the discriminator *D* is driven to maximize its ability to identify the generated fake images.

The specific architectural design choices in StratGAN are critical to the overall model performance. First, strided convolutions are adopted in the encoder, and downsampling is not implemented by a conventional max pooling layer, but is instead realized by convolutional layers with a 3×3 kernel and a stride of 2. In this design, the downsampling operation is converted into a learnable feature transformation process. Compared with max pooling, which discards substantial information in a fixed manner, strided convolutions enable dimensionality reduction while allowing the network to learn how waveform information that is most informative for the inversion task is preserved. For borehole radar data, which may contain weak reflections from distant strata or small fractures, this information retention capability is particularly important. Second, LeakyReLU is mainly used as the activation function. Compared with the standard ReLU, LeakyReLU retains a small nonzero gradient for negative inputs, which helps alleviate the neuron death problem and strengthens nonlinear representational capacity.

**Figure 2:**
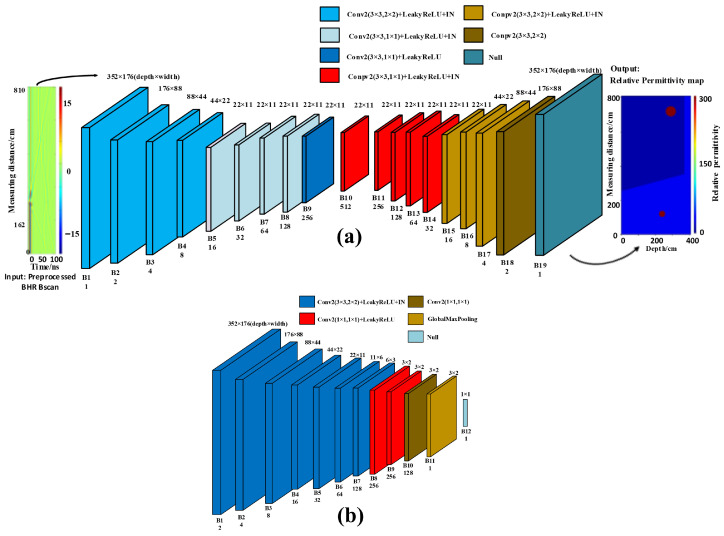
StratGAN network and overall architecture. (**a**) Generator architecture. (**b**) Discriminator architecture.

Notably, no activation function is applied at two locations in the generator. One location is the latent feature vector output by the last layer of the encoder, because omitting activation allows a broader numerical range to be retained and therefore improves feature representation. The other location is the final output layer of the decoder, because a physical quantity is directly predicted, namely the relative permittivity, and it should not be artificially constrained by the output range of an activation function. Finally, instance normalization (IN) is incorporated, and IN layers are appended after most convolutional layers in both the encoder and the decoder. IN normalizes each feature channel independently for each sample, thereby removing style-related information such as contrast and brightness variations across image instances. In GAN training, this design contributes to training stability and mitigates mode collapse. For borehole radar data, the signal amplitude may vary across logging intervals due to formation attenuation and instrument coupling, and IN enhances robustness to such amplitude variations. The generator architecture of StratGAN is detailed in Figure 2a.

The core idea of the StratGAN discriminator is not to output a single authenticity score for an entire input image, but to partition the image into multiple N×N patches and independently judge the authenticity of each patch, so that a discrimination map is finally produced. This local discrimination strategy is of particular relevance to geophysical inversion, because it encourages the generator to focus on producing locally realistic details and high-frequency textures. For borehole radar permittivity inversion, this means that the network is guided to generate stratified structures with clear and sharp boundaries, which constitute the key characteristics of layered media models. The discriminator architecture includes a convolutional feature extractor, a 1×1 convolutional classifier, and a global maximum pooling layer. The convolutional feature extractor is constructed by a series of convolutional layers, through which the spatial resolution of feature maps is progressively reduced while higher-level discriminative features are extracted. After feature extraction, a 1×1 convolutional layer is used for classification instead of a conventional fully connected layer. This design preserves the spatial structure of the feature space while efficiently performing linear combinations and dimensionality reduction along the channel dimension, and it is functionally equivalent to applying a small fully connected network at each patch location. The final output layer of the discriminator is implemented as a global maximum pooling layer.

However, it should be clarified that the patch-based discrimination adopted in this study does not correspond to a manually predefined fixed cropped patch size, but should instead be understood as an effective local discrimination scale that is implicitly determined by the discriminator architecture. Specifically, a discrimination map is formed in the spatial domain through multilayer convolution and progressive downsampling, and local authenticity is evaluated at different spatial locations, so that the generated results are constrained in a structured manner. Such a design allows the model to move beyond a single image level real or fake judgment and to simultaneously focus on layered interfaces, local anomalies, and high-frequency textures, all of which are particularly important for borehole radar permittivity inversion. From a mechanistic perspective, this local discrimination strategy represents an empirical compromise between local detail recovery and global stratified consistency. A relatively large effective local discrimination scale is beneficial for enhancing interface continuity, suppressing local artifacts, and improving structural fidelity. However, for extremely thin layers, small fractures, and deep weak anomalies with low signal-to-noise ratio, the local response may be partially diluted by broader background information. Therefore, the adopted patch-based discrimination is more appropriately described as a large receptive field local discrimination mechanism rather than a simple fixed small patch scheme. The discriminator architecture of StratGAN is detailed in Figure 2b.

During training, an alternating optimization strategy is adopted for the generator and the discriminator, with an update ratio of 1:2. Specifically, the generator parameters are fixed while the discriminator is updated for two steps, after which the discriminator parameters are fixed and the generator is updated for one step. RMSprop is used as the optimizer for both networks. The learning rates of the generator and the discriminator are set to $1.25\times 10^{-5}$ and $1.25\times 10^{-4}$, respectively, and the momentum parameter is set to 0.9 for both. The input size is fixed at 352×176, the batch size is set to 4, and the total number of training epochs is set to 720. In addition, a plateau-based learning-rate scheduling strategy is introduced. When the training metric is not further improved for 16 consecutive epochs, the learning rates of both the generator and the discriminator are reduced to 0.6 times their previous values. The model with the best overall performance is finally selected as the final model. It should be noted that these hyperparameter settings are determined as an empirical compromise after jointly considering adversarial training stability, GPU memory constraints, and the relatively high resolution of borehole radar Bscan data. Their purpose is to achieve a reasonable balance among convergence stability, computational cost, and inversion accuracy, rather than to serve as universally optimal fixed settings for all borehole radar inversion tasks.

The optimization objective is composed of two terms, namely a conditional adversarial loss and an $L_{1}$ loss, and it is expressed as follows:

(3) $$\begin{array}{cc} G^{*}\; & =\arg\min_{G}\max_{D}\;E_{d,\varepsilon_{r}^{\mathrm{ref}}}\;\left[\log D\;\left(d,\varepsilon_{r}^{\mathrm{ref}}\right)\right]+E_{d,\mathrm{rand}}\;\left[\log\;\left(1-D\;\left(d,G(d,\mathrm{rand})\right)\right)\right]\\ \; & \;+\lambda \left|\varepsilon_{r}^{\mathrm{ref}}-G\left(d,\mathrm{rand}\right)\right| \end{array}$$

In Equation (3), the random noise rand is introduced to encourage the network to generate more diverse images, whereas the influence on image quality is limited. The $L_{1}$ loss is adopted to reduce the blurring effect in the generated images. When the input noise and the $L_{1}$ term are removed, the objective is simplified to the standard conditional adversarial loss, which is written as:

(4) $$\begin{array}{cc} G^{*}\; & =\arg\min_{G}\max_{D}\;E_{d,\varepsilon_{r}^{\mathrm{ref}}}\;\left[\log D\;\left(d,\varepsilon_{r}^{\mathrm{ref}}\right)\right]+E_{d}\;\left[\log\;\left(1-D\;\left(d,G(d)\right)\right)\right] \end{array}$$

Within the adversarial training framework, the discriminator *D* is trained to identify the fake images produced by the generator *G* as accurately as possible, whereas the generator *G* is trained to produce outputs that cannot be distinguished from the real images. The primary objective of Equation (4) is to minimize the Jensen–Shannon divergence. However, a critical limitation of the Jensen–Shannon divergence is encountered when the support sets of the real data distribution $\varepsilon_{r}^{\mathrm{ref}}$ and the generated data distribution G(d) do not overlap in a high-dimensional space. Under this condition, updating the generator *G* tends to yield gradients that approach zero, which triggers the vanishing gradient problem in conditional GAN training. In borehole radar media inversion, the permittivity map is a high-dimensional vector, and real geological structures such as fractures and cavities occupy only an extremely sparse and discontinuous manifold in this space. In this case, the generated images are likely to be far from the real data manifold during the early stage of training, so that the two distributions are nearly non-overlapping, and effective gradient signals cannot be provided by the Jensen–Shannon divergence.

To address this issue, the Wasserstein distance is introduced. The Wasserstein distance, also referred to as the earth mover distance, measures the minimum cost required to transport one distribution into another. Even when two distributions do not overlap, the Wasserstein distance is still able to provide smooth and continuous gradients, which makes it well suited for handling high-dimensional sparse data distributions. When the Wasserstein distance is applied to StratGAN, the training loss is expressed as:

(5) $$\begin{array}{cc} G^{*}\; & =\arg\min_{G}\max_{{D,\;∥D∥}_{L}\leq 1}\;E_{d}\;\left[D\;\left(d,G(d)\right)\right]-E_{d,\varepsilon_{r}^{\mathrm{ref}}}\;\left[D\;\left(d,\varepsilon_{r}^{\mathrm{ref}}\right)\right]. \end{array}$$

Here, *G* denotes the generator and *D* denotes the discriminator. For the inversion of borehole radar Bscan data, *d* denotes the sampled scattering field that is used as the input. The generator output G(d) corresponds to the generated permittivity distribution $\varepsilon_{r}^{G}$, whereas $\varepsilon_{r}^{\mathrm{ref}}$ denotes the real permittivity distribution.

For the constraint ${∥D∥}_{L}\leq 1$ that is required to ensure model validity, the most primitive solution is to clip the parameters to a small range after each update. However, this approach introduces discontinuous clipping and causes pronounced oscillations during training. By adopting the loss formulation of WGAN-GP, the constraint ${∥D∥}_{L}\leq 1$ is converted into a gradient penalty term and is incorporated into the loss as a regularization component, so that the improved loss function is expressed as:

(6) $$\begin{array}{cc} G^{*}\; & =\arg\min_{G}\max_{D}\;E_{d,\varepsilon_{r}^{G}}\;\left[D\;\left(d,\varepsilon_{r}^{G}\right)\right]-E_{d,\varepsilon_{r}^{\mathrm{ref}}}\;\left[D\;\left(d,\varepsilon_{r}^{\mathrm{ref}}\right)\right]\\ \; & \;-\lambda \;E_{d,\varepsilon_{r}^{I}}\;\left[{\left({∥\nabla_{\left(d,\varepsilon_{r}^{I}\right)}D\;\left(d,\varepsilon_{r}^{I}\right)∥}_{2}-1\right)}^{2}\right] \end{array}$$

Here, $\varepsilon_{r}^{I}$ represents the distribution over the full space between the generated permittivity distribution $\varepsilon_{r}^{G}$ and the real distribution $\varepsilon_{r}^{\mathrm{ref}}$. In this study, $\varepsilon_{r}^{I}$ is approximated by a random interpolation between the real permittivity map $\varepsilon_{r}^{\mathrm{ref}}$ and the generated permittivity map $\varepsilon_{r}^{G}$. As shown in Equation (7), ω is sampled from a uniform distribution on [0,1], so that $\varepsilon_{r}^{I}$ is constructed as a random interpolation of $\varepsilon_{r}^{G}$ and $\varepsilon_{r}^{\mathrm{ref}}$, thereby approximating the full space distribution between $\varepsilon_{r}^{G}$ and $\varepsilon_{r}^{\mathrm{ref}}$. Gradient smoothing is applied to $\varepsilon_{r}^{I}$ to ensure that the gradients in the transition region are smooth.

(7) $$\varepsilon_{r}^{I}=\omega \;\varepsilon_{r}^{\mathrm{ref}}+\left(1-\omega \right)\;\varepsilon_{r}^{G}$$

The variable ω is randomly drawn from a uniform distribution between 0 and 1. This gradient smoothing operation ensures that the gradient in the transition region between the two distributions is smooth. From a patch-based perspective, a strong correspondence between the inputs within different patches is desired by the network. When such correspondence is strengthened, the learning complexity of the discriminator is reduced. Considering electromagnetic wave propagation, echoes received at different times undergo different levels of attenuation, which significantly weakens the correspondence between $d^{\left(m\right)}$ and $\varepsilon_{r}^{\mathrm{ref}(m)}$ within a patch. By replacing the input condition $\varepsilon_{r}^{\mathrm{ref}(m)}$, interpolation and sampling operations are reduced, and the correspondence between the inputs is enhanced. Following this idea, the optimized loss function of StratGAN is formulated as follows:

(8) $$\begin{array}{cc} G^{*}\; & =\arg\min_{G}\max_{D}\;E_{\varepsilon_{r}^{\mathrm{ref}},\varepsilon_{r}^{G}}\;\left[D\;\left(\varepsilon_{r}^{\mathrm{ref}},\varepsilon_{r}^{G}\right)\right]-E_{\varepsilon_{r}^{\mathrm{ref}},\varepsilon_{r}^{\mathrm{ref}}}\;\left[D\;\left(\varepsilon_{r}^{\mathrm{ref}},\varepsilon_{r}^{\mathrm{ref}}\right)\right]\\ \; & \;-\lambda \;E_{\varepsilon_{r}^{\mathrm{ref}},\varepsilon_{r}^{I}}\;\left[{\left({∥\nabla_{\left(\varepsilon_{r}^{\mathrm{ref}},\varepsilon_{r}^{I}\right)}D\;\left(\varepsilon_{r}^{\mathrm{ref}},\varepsilon_{r}^{I}\right)∥}_{2}-1\right)}^{2}\right] \end{array}$$

Under this new loss function, the discriminator is treated as a metric that evaluates the proximity between the generated image $\varepsilon_{r}^{G}$ and the real image $\varepsilon_{r}^{\mathrm{ref}}$. Typically, the mean value over all *M* patches is used as the discriminator output, and the corresponding optimization term of the loss function is expressed as:

(9) $$\begin{array}{cc} G^{*}\; & =\arg\min_{G}\max_{D}\;E_{\varepsilon_{r}^{\mathrm{ref}},\varepsilon_{r}^{G}}\;\left[\sum_{m=1}^{M}D\;\left(\varepsilon_{r}^{\mathrm{ref}(m)},\varepsilon_{r}^{G(m)}\right)\right]-E_{\varepsilon_{r}^{\mathrm{ref}},\varepsilon_{r}^{\mathrm{ref}}}\;\left[\sum_{m=1}^{M}D\;\left(\varepsilon_{r}^{\mathrm{ref}(m)},\varepsilon_{r}^{\mathrm{ref}(m)}\right)\right]\\ \; & \;-\lambda \;E_{\varepsilon_{r}^{\mathrm{ref}},\varepsilon_{r}^{I}}\;\left[{\left({∥\nabla_{\left(\varepsilon_{r}^{\mathrm{ref}},\varepsilon_{r}^{I}\right)}\sum_{m=1}^{M}D\;\left(\varepsilon_{r}^{\mathrm{ref}(m)},\varepsilon_{r}^{I(m)}\right)∥}_{2}-1\right)}^{2}\right] \end{array}$$

After the loss is decomposed into an optimization term and a regularization term, it is written as follows:

(10) v(G,D)=O(G,D)+R(G,D)

The optimization term is given by:

(11) $$\begin{array}{cc} O(G,D)= & E_{\varepsilon_{r}^{\mathrm{ref}},\varepsilon_{r}^{G}}\;\left[\sum_{m=1}^{M}D\;\left(\varepsilon_{r}^{\mathrm{ref}(m)},\varepsilon_{r}^{G(m)}\right)\right]-E_{\varepsilon_{r}^{\mathrm{ref}},\varepsilon_{r}^{\mathrm{ref}}}\;\left[\sum_{m=1}^{M}D\;\left(\varepsilon_{r}^{\mathrm{ref}(m)},\varepsilon_{r}^{\mathrm{ref}(m)}\right)\right] \end{array}$$

As indicated by the averaging operation in Equation (11), the network is encouraged to focus on the overall image level error, which causes the model to preferentially fit large background regions that are easier to learn while neglecting small target regions that are more challenging when scenes contain both large background structures and small targets. In borehole radar surveys, accurate inversion of subtle geological anomalies such as small fractures, cavities, or localized water bearing zones is essential. To address this issue, the averaging operation in the loss is replaced by global max pooling. The corresponding mathematical formulation is written as:

(12) $$\begin{array}{cc} O(G,D)= & E_{\varepsilon_{r}^{\mathrm{ref}},\varepsilon_{r}^{G}}\;\left[\min_{m}D\;\left(\varepsilon_{r}^{\mathrm{ref}(m)},\varepsilon_{r}^{G(m)}\right)\right]-E_{\varepsilon_{r}^{\mathrm{ref}},\varepsilon_{r}^{\mathrm{ref}}}\;\left[\max_{m}D\;\left(\varepsilon_{r}^{\mathrm{ref}(m)},\varepsilon_{r}^{\mathrm{ref}(m)}\right)\right] \end{array}$$

In practice, global max pooling can be simplified by introducing a negative judgment before the discriminator output layer. The final optimized loss function is written as:

(13) $$\begin{array}{cc} O(G,D)= & -E_{\varepsilon_{r}^{\mathrm{ref}},\varepsilon_{r}^{G}}\;\left[\min_{m}-D\;\left(\varepsilon_{r}^{\mathrm{ref}(m)},\varepsilon_{r}^{G(m)}\right)\right]-E_{\varepsilon_{r}^{\mathrm{ref}},\varepsilon_{r}^{\mathrm{ref}}}\;\left[\max_{m}D\;\left(\varepsilon_{r}^{\mathrm{ref}(m)},\varepsilon_{r}^{\mathrm{ref}(m)}\right)\right] \end{array}$$

The key idea of this modification is to force the network to focus on the patches with the largest errors. These high-error regions are often associated with small targets that are difficult to fit. By penalizing the maximum error, the model is guided to prioritize learning and improving the inversion accuracy for these small targets, which better matches practical borehole radar applications that require the detection of subtle anomalies. The regularization term is expressed as:

(14) $$\begin{array}{cc} R(G,D)= & -\lambda \;E_{\varepsilon_{r}^{\mathrm{ref}},\varepsilon_{r}^{I}}\;\left[{\left({∥\nabla_{\left(\varepsilon_{r}^{\mathrm{ref}},\varepsilon_{r}^{I}\right)}\sum_{m=1}^{M}D\;\left(\varepsilon_{r}^{\mathrm{ref}(m)},\varepsilon_{r}^{I(m)}\right)∥}_{2}-1\right)}^{2}\right] \end{array}$$

As shown in Equation (14), a regularization term based on averaging tends to be insensitive to small targets in large background and small target scenarios, because global errors dominate and large background regions are preferentially fitted. Therefore, the regularization term is modified in StratGAN by replacing the averaging operation with global max pooling, so that the response to locally salient features, namely small targets, is strengthened. The modified regularization term is expressed as:

(15) $$\begin{array}{cc} R(G,D)\; & =-\lambda \;E_{\varepsilon_{r}^{\mathrm{ref}},\varepsilon_{r}^{I}}\;\left[{\left({∥\nabla_{\left(\varepsilon_{r}^{\mathrm{ref}},\varepsilon_{r}^{I}\right)}\max_{m}\;\left(-D\;\left(\varepsilon_{r}^{\mathrm{ref}(m)},\varepsilon_{r}^{I(m)}\right)\right)∥}_{2}-1\right)}^{2}\right]\\ \; & \;+\;\left[{\left({∥\nabla_{\left(\varepsilon_{r}^{\mathrm{ref}},\varepsilon_{r}^{I}\right)}\max_{m}\;\left(-D\;\left(\varepsilon_{r}^{\mathrm{ref}(m)},\varepsilon_{r}^{I(m)}\right)\right)∥}_{2}-1\right)}^{2}\right] \end{array}$$

Finally, the StratGAN loss function that incorporates both the regularization term and global max pooling is written as Equation (16). In Equation (16), the coefficient λ is used solely as the weight of the gradient-penalty regularization term. Its primary role is to regularize the discriminator so that the Lipschitz constraint required by WGAN-GP is better satisfied, thereby improving the stability of adversarial training. When λ is too small, discriminator regularization becomes insufficient, the training process becomes more prone to oscillation, and the structural constraint signal also becomes unstable. In contrast, when λ is too large, the discriminator may be overly constrained, which weakens its discriminative ability and consequently reduces the effectiveness of the guidance provided to the generator. Therefore, the coefficient in Equation (16) mainly affects training stability, whereas its influence on boundary sharpness and quantitative permittivity accuracy is reflected indirectly through the overall balance between adversarial optimization and regularization. At the same time, structural clarity and quantitative accuracy are jointly influenced by the adversarial term and the data-consistency term. A stronger data-consistency constraint is usually beneficial for improving the quantitative approximation of permittivity values, but it also tends to produce smoother results. By contrast, a stronger adversarial constraint is beneficial for recovering sharper boundaries and richer structural details, although excessively strong adversarial supervision may introduce local spurious structures. Therefore, the corresponding weight is more appropriately regarded as an empirically selected tradeoff parameter for the current task setting and noise condition, rather than as a universally optimal constant applicable to all scenarios.

(16) $$\begin{array}{cc} G^{*}=\arg\min_{G}\max_{D}\; & -E_{\varepsilon_{r}^{\mathrm{ref}},\varepsilon_{r}^{G}}\;\left[\max_{m}\;\left(-D\;\left(\varepsilon_{r}^{\mathrm{ref}(m)},\varepsilon_{r}^{G(m)}\right)\right)\right]-E_{\varepsilon_{r}^{\mathrm{ref}},\varepsilon_{r}^{\mathrm{ref}}}\;\left[\max_{m}D\;\left(\varepsilon_{r}^{\mathrm{ref}(m)},\varepsilon_{r}^{\mathrm{ref}(m)}\right)\right]\\ \; & -\lambda \;E_{\varepsilon_{r}^{\mathrm{ref}},\varepsilon_{r}^{I}}\;\left[{\left({∥\nabla_{\left(\varepsilon_{r}^{\mathrm{ref}},\varepsilon_{r}^{I}\right)}\max_{m}\;\left(-D\;\left(\varepsilon_{r}^{\mathrm{ref}(m)},\varepsilon_{r}^{I(m)}\right)\right)∥}_{2}-1\right)}^{2}\right]\\ \; & +\;\left[{\left({∥\nabla_{\left(\varepsilon_{r}^{\mathrm{ref}},\varepsilon_{r}^{I}\right)}\max_{m}D\;\left(\varepsilon_{r}^{\mathrm{ref}(m)},\varepsilon_{r}^{I(m)}\right)∥}_{2}-1\right)}^{2}\right] \end{array}$$

## 3. Numerical Experiments

### 3.1. Numerical Dataset Generation

**Figure 3:**
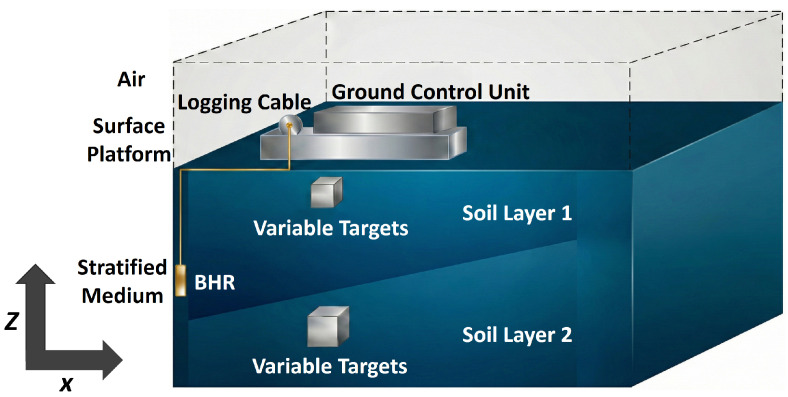
Schematic illustration of a typical modeling configuration for BHR target Bscan acquisition.

To construct a physically consistent and reproducible borehole radar (BHR) dataset, numerical simulations are performed using the open source finite difference time-domain (FDTD) software gprMax [37]. Considering computational efficiency and the demand for large-scale data generation, a two-dimensional vertical section is adopted in the simulation stage to approximate the equivalent three-dimensional sensing scenario, as illustrated in Figure 3.

The model is defined in a Cartesian coordinate system, and the computational domain is set to 4.17m×8.70m, where the horizontal radial direction is denoted by *x* and the vertical depth direction is denoted by *z*. The spatial discretization step is set to Δ=0.01m. To emulate typical sedimentary formations, the background is configured as a two-layer stratified medium, where the relative permittivity $\varepsilon_{r}$ of the upper layer (z>2.85m) and the lower layer (z≤2.85m) is set to 4.0 and 16.0, respectively, and the conductivity σ of both layers is set to 0.0005S/m. To represent complex geological contacts, the lower layer medium is filled within a polygonal region bounded by the vertices (0.12,2.85), (3.8,3.85), and (3.8,2.85) in meters, so that an inclined transition zone is constructed. In addition, an air strip with relative permittivity 1 and conductivity 0S/m is placed at x∈[0.10m,0.12m] to equivalently represent the borehole cavity.

For data acquisition, a Ricker wavelet with a central frequency of 86MHz is used as the excitation source. A co-located transmitting and receiving Hertzian dipole polarized is positioned at the borehole center (x=0.11m) to approximate the omnidirectional radiation characteristic of practical borehole antennas. The antenna is scanned along the *z* axis from 0.30m upward with a step size of 0.06m. A total of 136 traces are collected for each profile to form a Bscan image. The recording time window is set to 150ns to ensure that effective reflections from interfaces and targets are fully captured.

To evaluate the generalization capability of the algorithm for subsurface anomalies such as cavities and fractures, a parameter randomization strategy is introduced, in which two targets are randomly embedded in each model. The target material is randomly selected from three representative media to cover high, moderate, and low permittivity contrasts, including steel as a strong reflector with $\varepsilon_{r}=300$ and $\sigma =10^{8}\;S/m$, water with $\varepsilon_{r}=81$ and σ=0.005S/m, and saturated clay with $\varepsilon_{r}=45$ and σ=0.001S/m. The geometric shape of the targets, including circular, rectangular, and triangular types, as well as their size and location, are randomly assigned, conditioned on the stratigraphic layer. For circular targets, the radius is sampled from r∈[0.1m,0.35m], the radial position of the center is sampled from x∈[1.0m,3.0m], and the depth is randomly distributed either in the upper layer with z∈[5.5m,7.5m] or in the lower layer with z∈[1.0m,2.5m]. For rectangular and triangular targets, both the base length and the height are randomly sampled from [0.1m,0.35m], the *x* coordinate of the lower left corner is sampled from x∈[0.8m,2.8m], and the depth is randomly assigned either in the upper layer with z∈[5.3m,7.3m] or in the lower layer with z∈[0.3m,2.3m].

Based on the above parameterized simulations, a total of 5400 paired samples that cover multiple geometric patterns and electromagnetic properties are generated. Each pair consists of one preprocessed Bscan image used as the network input and the corresponding permittivity distribution model used as the ground truth label. Finally, the dataset is randomly split into a training set of 4320 samples and a test set of 1080 samples with a ratio of 8:2, which are used for neural network parameter optimization and independent evaluation of inversion accuracy, respectively.

It should be noted that the present dataset is constructed primarily to validate the effectiveness of StratGAN under controlled stratified medium conditions, rather than to fully reproduce all complex physical processes encountered in real field borehole radar environments. Although fixed background permittivity values are adopted and the numerical simulations are based on a two-dimensional FDTD framework, a certain degree of interface geometric variation and target contrast diversity is still introduced through inclined transitional interfaces and the randomization of target shape, position, size, and electromagnetic properties. However, such controlled modeling still cannot sufficiently cover key field factors such as spatially varying conductivity, borehole wall roughness, borehole fluid, antenna coupling, strong clutter, and three-dimensional heterogeneous scattering. Therefore, the present results are more appropriately interpreted as a stage-wise validation of the proposed method in simplified yet representative stratified scenarios. It should also be emphasized that the present study does not fall within the strict scope of extreme low sample learning. Although the data scale remains limited in comparison with large-scale annotated tasks in computer vision, and although geophysical labels are costly to obtain, 5400 pairs of simulated samples cannot yet be regarded as a highly scarce training set. Accordingly, the contribution of StratGAN is more accurately understood as an improvement in the stability and robustness of borehole radar inversion under relatively limited and label-expensive data conditions, rather than as a complete solution to extremely small sample learning. Further investigation is still required to assess inversion performance and generalization under more severely limited data conditions.

### 3.2. Result Analysis

#### 3.2.1. Inversion Simulation Case Study of a Cylindrical Anomaly (Circular Target) in Stratified Media

**Figure 4:**
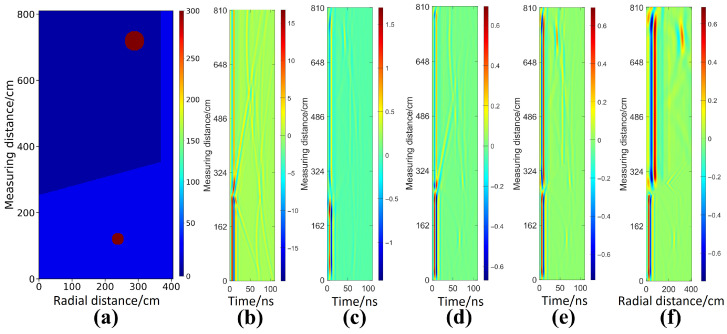
Comparison of simulated data and migration imaging for a circular target filled with steel. (**a**) Ground truth relative permittivity map in the investigation region. (**b**) Corresponding preprocessed Bscan of borehole radar. (**c**) Migration result under a global velocity assumption of 0.15m/ns. (**d**) Migration result under a global velocity assumption of 0.075m/ns. (**e**) Imaging profile obtained by joint migration. (**f**) Imaging profile obtained by the enhanced migration with migration and time depth joint conversion.

In this section, inversion tests are conducted on cylindrical anomalies with different permittivity contrasts to evaluate the imaging performance of each model for representative targets. A two-layer medium with background relative permittivities of 4 and 16 and an embedded steel target is considered as an example. Figure 4a,b present the ground truth permittivity distribution and the corresponding raw Bscan profile, respectively, where the latter clearly exhibits strong diffractive hyperbolas associated with the targets located in the upper and lower layers. Owing to the dual-velocity characteristics in stratified media, conventional single-velocity migration struggles to simultaneously achieve target focusing and accurate spatial positioning. As shown in Figure 4c,d, adopting a single high-velocity or low-velocity model leads to under-migrated or over-migrated diffraction artifacts for targets in the mismatched layer, accompanied by pronounced depth misplacement. Conventional time-domain joint migration in Figure 4e improves wavefield focusing, yet geometric distortions induced by cross-layer propagation are not corrected. In contrast, the enhanced migration approach in Figure 4f decouples time-domain focusing from space domain time depth conversion, and the electromagnetic kinematics in heterogeneous media are therefore followed more faithfully, so that the true spatial location and coarse geometry of the target are recovered more accurately. However, such migration-based methods remain essentially limited to qualitative structural reconstruction, and quantitative estimation of target permittivity is not provided. This limitation further highlights the necessity of introducing deep learning models in this study to achieve high-accuracy quantitative inversion of electromagnetic parameters.

**Figure 5:**
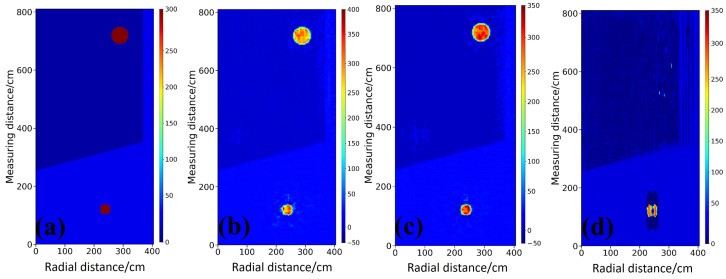
Comparison of permittivity inversion results for circular targets filled with steel. (**a**) Ground truth permittivity scenario. (**b**) StratGAN. (**c**) CNN. (**d**) Permittivity inversion result of GPRNet.

For the steel sphere targets with high-permittivity contrast, the inversion results of StratGAN in Figure 5b and CNN in Figure 5c, where the network structure is consistent with the StratGAN generator, are in good agreement with the ground truth model in Figure 5a in terms of target shape, location, and boundary sharpness, and both circular targets are reconstructed clearly. In particular, the boundary of the upper target is rendered sharper by StratGAN, which is attributed to the adversarial learning framework that tends to generate images with richer high-frequency details and thus mitigates the smoothing effect caused by conventional pixelwise loss functions. By contrast, as shown in Figure 5d, the inversion produced by GPRNet [28] is of the lowest quality, where severe target deformation is observed and the stratified interface in the background is distorted in a manner that is inconsistent with physical expectations, indicating that the propagation behavior of electromagnetic waves in stratified media is not effectively learned.

**Figure 6:**
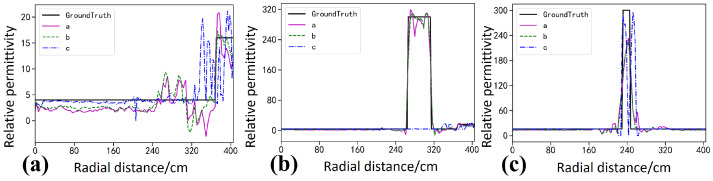
Comparison of Ascan-based inversion results for circular targets filled with steel. Ground Truth denotes the reference permittivity. Curves a–c correspond to StratGAN, CNN, and GPRNet, respectively. (**a**) Background permittivity. (**b**) Permittivity of the upper target. (**c**) Permittivity of the lower target.

The Ascan profiles in Figure 6 provide a more detailed quantitative comparison. Along the profiles intersecting the targets, as shown in Figure 6b,c, the permittivity curve of StratGAN, shown as the pink curve a, matches the ground truth, shown as the black curve, most closely, and both the peak amplitude and the width of the high-permittivity response are reproduced accurately. The CNN result, shown as the green dashed curve b, is similar to that of StratGAN, yet a slightly lower peak is observed for the lower target, which indicates a larger quantitative bias. The GPRNet result, shown as the blue dash dotted curve c, deviates substantially from the ground truth, and the inversion of the upper target is even abandoned in Figure 6b, where the curve becomes nearly flat and only the background permittivity is fitted, which provides direct evidence of inversion failure.

The inversion results are further quantified, and the metrics are listed in Table 1. The quantitative results corroborate the visual inspection. StratGAN achieves the best performance in terms of MAE with a value of 2.7268 and $R^{2}$ with a value of 0.9431, which indicates superior quantitative accuracy and stronger explanatory power for the model variance. It is noteworthy that the SSIM of GPRNet is slightly higher than that of StratGAN with a value of 0.9246, whereas its $R^{2}$ is only 0.1129. This result suggests that the inversion produced by GPRNet lacks effective statistical correlation with the ground truth. The relatively high SSIM score is likely dominated by fitting the background structure, while the inversion of the key targets is not successful.

#### 3.2.2. Inversion Simulation Case Study of Planar and Block-Shaped Anomalies in Stratified Media

**Figure 7:**
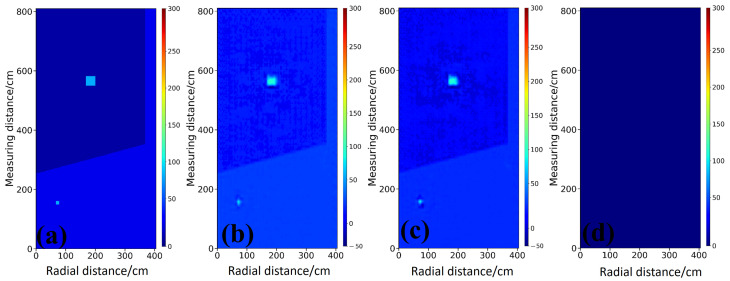
Comparison of inversion results for rectangular targets filled with water. (**a**) Ground truth permittivity scenario. (**b**) Permittivity inversion result of StratGAN. (**c**) Permittivity inversion result of CNN. (**d**) Permittivity inversion result of GPRNet.

As shown in Figure 7, under the medium contrast water filled scenario, StratGAN maintains good geometric fidelity and reconstructs two rectangular targets, as illustrated in Figure 7b. A similar performance is observed for CNN in Figure 7c. By contrast, as shown in Figure 7d, GPRNet fails completely, and no target-related information is preserved in the output image.

**Figure 8:**
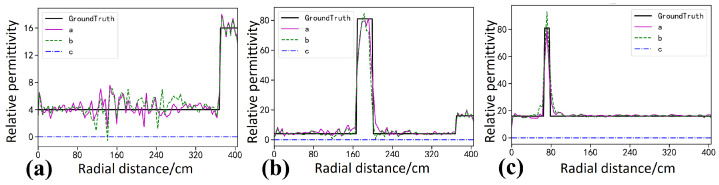
Comparison of Ascan-based inversion results for rectangular targets filled with water. Ground Truth denotes the reference permittivity. Curves a–c correspond to StratGAN, CNN, and GPRNet, respectively. (**a**) Background permittivity comparison. (**b**) Permittivity comparison for the upper target. (**c**) Permittivity comparison for the lower target.

The Ascan profiles in Figure 8 provide a more detailed quantitative comparison. Along the profiles intersecting the targets in Figure 8b,c, the permittivity values estimated by StratGAN, shown as the pink curve a, match the ground truth, shown as the black curve, most closely, and both the peak amplitude and the width of the high-permittivity response are reproduced. The CNN result, shown as the green dashed curve b, is similar to that of StratGAN, yet the peak at the target location is overestimated. The GPRNet result, shown as the blue dash dotted curve c, deviates substantially from the ground truth and effectively abandons the inversion of target permittivity, because the background permittivity is directly fitted.

The metrics in Table 2 corroborate the visual inspection. StratGAN achieves the best performance in terms of MAE, $R^{2}$, SSIM, and MSSIM, which demonstrates its superior inversion interpretability. It is noteworthy that the $R^{2}$ of GPRNet is only −1.6655, which indicates that its inversion results lack effective statistical correlation with the ground truth. The low SSIM value further suggests that the background structure is also weakly fitted in this case, while the inversion of the key targets is even more unsuccessful.

#### 3.2.3. Inversion Simulation Case Study of Irregular Triangular Anomalies in Stratified Media

**Figure 9:**
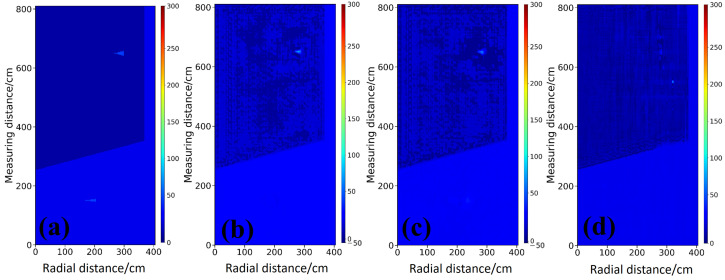
Compares inversion results for a triangular target (saturated clay). (**a**) shows the ground truth permittivity model. (**b**–**d**) show the permittivity inversion results of StratGAN, CNN, and GPRNet, respectively.

As shown in Figure 9, the challenge of inverting irregular shapes is further increased under the low-contrast saturated clay scenario. As shown in Figure 9b, the inversion produced by StratGAN achieves the best agreement in both morphology and location, and the outline of the upper triangular target is still approximately delineated. As shown in Figure 9c, the CNN result appears slightly blurred. It is worth noting that both StratGAN and CNN perform poorly when inverting the lower target, which indicates that the performance of end-to-end models begins to reach a bottleneck under the dual challenges of low signal-to-noise ratio and irregular geometry. As shown in Figure 9d, GPRNet fails to reconstruct any target, and its output is almost indistinguishable from the background model.

**Figure 10:**
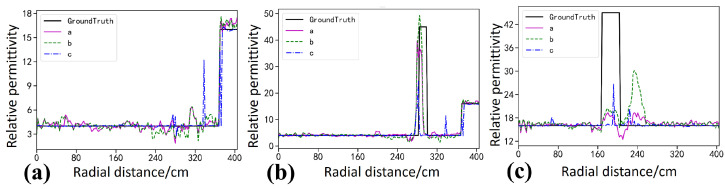
Compares Ascan permittivity inversion results for a triangular target (saturated clay). Ground Truth denotes the true permittivity. Curves a–c correspond to the Ascan inversion results of StratGAN, CNN, and GPRNet, respectively. (**a**) corresponds to the background, (**b**) corresponds to the upper target, and (**c**) corresponds to the lower target.

The Ascan profiles in Figure 10 provide a more detailed quantitative comparison. For the profiles crossing the targets in Figure 10b,c, the permittivity estimated by StratGAN (magenta curve a) agrees well with the ground truth (black curve, Ground Truth). The CNN result (green dashed curve b) exhibits a trend similar to that of StratGAN, whereas GPRNet (blue dash dotted curve c) deviates substantially from the ground truth. For example, in Figure 10b,c, when the target permittivity is inverted, the peak is excessively narrow and is accompanied by spurious ghosting artifacts.

It should be further noted that the degradation observed for low-contrast weak anomalies, especially for the deeper lower target, is not accidental, but is jointly caused by reduced physical observability and bias in the learning optimization process. For weak anomalies such as saturated clay, the permittivity contrast with the background medium is small, so the reflection coefficient is reduced and the effective scattering response is weakened. At the same time, deeper targets are subject to longer propagation paths and more complex cross-layer propagation processes, and are therefore more easily affected by refraction in stratified media, multipath propagation, and noise interference. On the other hand, weak anomalies usually occupy a small area, whereas the stratified background dominates the scene, so pointwise regression losses are more likely to drive the model to fit the background preferentially, thereby weakening sensitivity to local weak permittivity variations. Therefore, low contrast, deep, and geometrically irregular weak anomalies remain an important challenge for the current StratGAN framework.

As summarized in Table 3, the performance gap among the models is reduced for the triangular target inversion. In some cases, the metrics of GPRNet are improved, and a certain level of competitiveness is even observed in terms of MAE. However, from the visualization perspective, GPRNet is still more focused on inverting background information while the target is further neglected, and the $R^{2}$ value remains the lowest. Overall, StratGAN and CNN still exhibit higher $R^{2}$ and SSIM values in most cases, which indicates that their inversion results are more reliable and accurate in a global sense. The performances of StratGAN and CNN are very close and alternate in advantage, while StratGAN is typically superior in visual quality, which reflects the advantage of the StratGAN framework in generating high-frequency details.

To provide a final and statistically robust conclusion on the overall performance of the three models across the entire simulated dataset, the mean metrics over all 1080 test samples are computed in Table 4. The full dataset yields a decisive conclusion for the simulation study, namely that StratGAN achieves the best performance. StratGAN attains the best scores on all four evaluation metrics, and its lead in MAE, $R^{2}$, SSIM, and MSSIM demonstrates its overall superiority as a borehole radar inversion algorithm. The CNN exhibits stable performance. As a baseline that shares the same architecture as the StratGAN generator, the CNN demonstrates strong inversion capability, and its metrics are very close to those of StratGAN, which validates the effectiveness of deep convolutional networks for this type of image-to-image inversion problem. By contrast, GPRNet fails. The performance of GPRNet over the full test set is instead quite poor. Its mean $R^{2}$ is −0.0038, which is nearly zero and indicates that the data variance is not explained at all. Its MAE is more than three times that of StratGAN, and both SSIM and MSSIM are also substantially lower than those of the other two methods. These results confirm that the previously observed background fitting collapse of GPRNet is systematic rather than accidental. Therefore, GPRNet is not suitable for geophysical inversion tasks that require accurate detection of local anomalies under data-limited conditions.

## 4. Tests with Real Measurement Data

### 4.1. Dataset Collection and Experimental Setup

**Figure 11:**
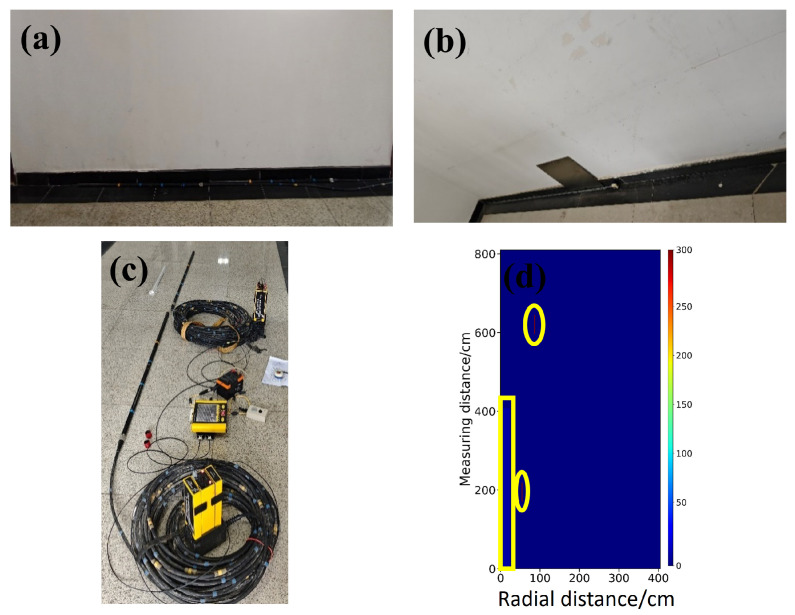
The in situ measurement scenario and detection configuration for the rectangular steel targets. (**a**,**b**) show photographs of the field measurement environment. (**c**) shows the commercial EKKO borehole radar system used in the experiment. (**d**) illustrates the acquisition profile. The physical model is composed of two background media, namely a wall ($\varepsilon_{r}=4$) and air ($\varepsilon_{r}=1$), and two steel plates are preset inside the wall as the detection targets. For ease of identification, the spatial locations of the upper and lower steel plates, as well as the main wall region, are marked in (**d**) using a yellow circle and a yellow rectangular box, respectively.

As shown in Figure 11, the measurement is conducted in a controlled indoor environment to emulate the scenario in which borehole radar detects targets behind a borehole wall. Figure 11a,b show photographs of the measurement site, Figure 11c shows the commercial EKKO borehole radar instrument, and Figure 11d shows a schematic of the survey profile. The experimental scene contains two background media, a wall ($\varepsilon_{r}=4$) and air ($\varepsilon_{r}=1$), and two steel plate targets are deployed. In Figure 11d, the positions of the upper and lower steel plates are indicated by yellow circles, while the wall region is highlighted by a yellow rectangular box.

### 4.2. Result Analysis

**Figure 12:**
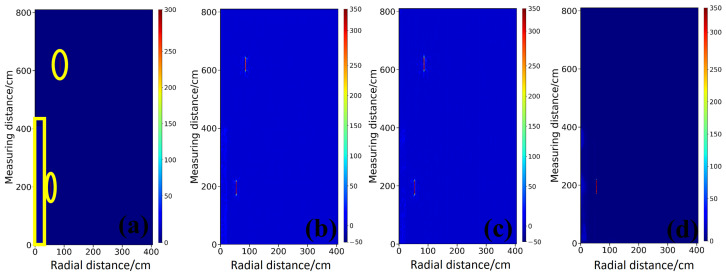
Compares the inversion results for the rectangular steel targets in the measured scene. (**a**) shows the ground truth permittivity model. (**b**–**d**) show the permittivity inversion results of StratGAN, CNN, and GPRNet, respectively. The physical model is composed of two background media, namely a wall ($\varepsilon_{r}=4$) and air ($\varepsilon_{r}=1$), and two steel plates are preset inside the wall as the detection targets. For ease of identification, the spatial locations of the upper and lower steel plates, as well as the main wall region, are marked in (**a**) using a yellow circle and a yellow rectangular box, respectively.

As shown in Figure 12, the real data contain complex factors such as instrument noise, antenna coupling, and environmental clutter, and a higher level of robustness is therefore required. As shown in Figure 12b, StratGAN yields the clearest and most accurate inversion under these conditions. The interface between the wall and the air layer is successfully reconstructed, and the steel plate targets are precisely localized, with sharp boundaries and minimal artifacts. These results indicate that the physical patterns learned from the simulated data are generalized well to the real measurement domain. As shown in Figure 12c, the CNN result is also satisfactory, but the target boundaries are slightly blurred and noticeable distortions occur in the wall region, which suggests a weaker adaptability to domain shift. As shown in Figure 12d, GPRNet produces the lowest-quality inversion, with severe distortion in the reconstructed wall, a weak response for the upper target, and overall degraded interpretability, which further indicates its vulnerability when non-ideal data are encountered.

**Figure 13:**
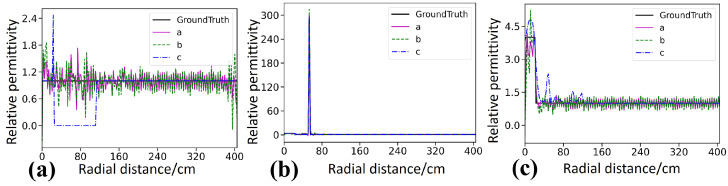
Compares Ascan inversion results for the rectangular steel targets in the measured scene. Ground Truth denotes the true permittivity. Curves a–c correspond to the Ascan inversion results of StratGAN, CNN, and GPRNet, respectively. (**a**) corresponds to the background, (**b**) corresponds to the upper target, and (**c**) corresponds to the lower target.

As indicated by the Ascan profiles in Figure 13, StratGAN (magenta curve a) achieves the closest agreement with the ground truth (black curve) in terms of wall thickness, target location, and permittivity magnitude, and its profile is smooth and accurate. The CNN result (green dashed curve b) exhibits a larger deviation from the ground truth, whereas GPRNet (blue dash dotted curve c) deviates substantially. In particular, the background permittivity is even fitted toward zero, and abundant ghosting artifacts are produced in the target regions.

The quantitative metrics in Table 5 further confirm the superiority of StratGAN. The highest scores are achieved in $R^{2}$ (0.9602), SSIM (0.9966), and MSSIM (0.9992). By contrast, the $R^{2}$ of GPRNet is only 0.4965, which is substantially lower than those of the other two models, indicating that its ability to explain the variance of data containing real noise and complexity is severely limited. This observation further suggests that the failure mode of GPRNet is not confined to simulated data, and that it is unreliable in practical applications.

The evaluation on the full measured dataset is summarized in Table 6, and the conclusion drawn from the simulation experiments is finally confirmed. StratGAN performs best in three key metrics, namely $R^{2}$, SSIM, and MSSIM, which demonstrates its favorable generalization capability and superior performance when transferring from simulated environments to the real physical world. These results indicate that StratGAN is not only theoretically advantageous but also achieves the highest reliability and accuracy in practice. The $R^{2}$ of GPRNet is only 0.5598, which is far lower than those of the other two models and again implies an insufficient capability to explain data variance in the presence of real noise and complexity. This further supports that the failure mode of GPRNet is not limited to simulations and remains unreliable in real applications.

Based on the combined numerical simulations and physical model experiments, the effectiveness of the proposed StratGAN is systematically validated for the borehole radar permittivity inversion task in stratified media. Compared with the standard CNN and GPRNet architectures, the performance improvement of StratGAN is primarily reflected in three aspects. First, quantitative accuracy is improved, as evidenced by lower mean absolute error (MAE) and higher coefficient of determination ($R^{2}$) in the test cases, which indicates that the prediction of permittivity magnitudes and background stratigraphic variations is more reliable. Second, structural fidelity is enhanced, as indicated by improved SSIM and MSSIM, and the geometry and sharp boundaries of anomalies are better recovered, which is important for accurate delineation of geological targets. Third, generalization robustness is strengthened, and stable imaging and inversion performance is maintained when different target shapes, property contrasts (for example steel, water, and saturated clay), and measured noise disturbances are encountered.

The comparative experiments further reveal a specific failure mode that is likely to occur when conventional deep learning models are applied to data-limited and complex geophysical inversions, namely background fitting collapse. For GPRNet in particular, the output tends to converge toward the background stratigraphy while small-scale anomalies are neglected in some test cases. This behavior is mainly induced by standard pixelwise loss functions under severe class imbalance between background pixels and target pixels. The network is guided to fit the dominant background region to reduce the global loss rapidly, and the optimization is driven toward a local optimum corresponding to a trivial solution that predicts only the background. This finding suggests that directly transferring generic computer vision architectures to geophysical tasks that are centered on anomaly detection involves a systematic risk of underfitting.

It should be noted that the conclusions of this study should be restricted to the present task setting of two-dimensional borehole radar permittivity inversion in stratified media. The available evidence indicates that StratGAN achieves superior overall performance relative to the architecture-matched CNN and GPRNet, but it is still insufficient to demonstrate universally superior performance over all advanced GPR inversion networks. More systematic comparisons with additional models under a unified task setting, a unified retraining protocol, and a unified evaluation framework are still required. From a mechanistic perspective, the advantage of StratGAN is derived mainly from the structural regularization provided by conditional adversarial learning. The discriminator is able to implicitly learn the continuity of stratified structures, the characteristics of target boundaries, and the plausibility of anomaly occurrence in the true permittivity distribution, so that smooth pseudo-solutions that fit only the background are suppressed, the problem of background fitting collapse is alleviated, and structural recovery is improved. At the same time, the engineering utility of StratGAN is reflected in the tradeoff between offline training cost and online inference efficiency. Although higher computational cost is incurred during training because alternating optimization of the generator and discriminator is required and WGAN GP regularization is introduced, end-to-end inversion is completed during deployment through only a single forward pass, and rapid interpretation potential is therefore retained.

It should also be emphasized that the proposed method remains, in essence, a data-driven encoder–decoder inversion model. Hard physical constraints such as Maxwell equation residuals, electromagnetic propagation consistency, and the permittivity velocity relation are not explicitly incorporated. Its physical plausibility is supported mainly by physically consistent FDTD data and the statistical structural priors learned by the adversarial discriminator. Therefore, locally unreasonable outputs may still be produced under out-of-distribution samples, strong noise, and complex field disturbances. In addition, the present study is confined to two-dimensional profile inversion and validation using a controlled indoor physical model, and it cannot yet be regarded as equivalent to complete three-dimensional geological characterization or field-scale engineering validation. The current results are more appropriately interpreted as stage-wise evidence for practical feasibility. Future work is directed toward systematic sensitivity analyses of physics data collaborative constraints, weak anomaly enhancement strategies, multiscale local discrimination mechanisms, and key factors such as optimizer type, batch size, learning rate scheduling, training epochs, and patch discrimination scale. Further extension toward three-dimensional settings and more complex field borehole radar data is also pursued.

## 5. Conclusions and Future Work

To address the severe ill posedness of borehole radar permittivity inversion in stratified media under data-limited conditions, limited observation aperture, and strong noise interference, a purely data-driven inversion paradigm termed StratGAN is proposed based on a conditional generative adversarial network. Through adversarial training between a generator and a discriminator, implicit statistical regularities embedded in radar waveform data are exploited to construct an effective regularization constraint. Without explicitly introducing physical priors, StratGAN establishes an end-to-end nonlinear mapping from waveforms to permittivity distributions, thereby alleviating the over-smoothing and boundary blurring that are commonly induced by conventional pixelwise regression methods.

Systematic evaluations on simulated and measured datasets across multiple representative geological models indicate that StratGAN achieves overall superior performance to CNN and GPRNet baselines in several quantitative metrics, including MAE, $R^{2}$, and SSIM, and demonstrates favorable generalization to varying target morphologies, property contrasts, and mixed noise. In particular, in the measured data validation, the coefficient of determination $R^{2}$ reaches 0.9533, which is substantially higher than that of GPRNet (0.5598), and high-fidelity interlayer structures and boundary continuity are reconstructed. In addition, a failure mode termed background fitting collapse is identified and defined for data-limited and strongly class-imbalanced settings, in which the network overfits the background and excessively smooths subtle anomalies. By leveraging adversarial learning of the global structural distribution, StratGAN avoids this local optimum trap to a certain extent and improves the reconstruction accuracy for small targets.

Overall, favorable quantitative permittivity inversion capability is demonstrated by StratGAN under simulated and controlled measured conditions, and a feasible deep learning solution is provided for high-resolution interpretation of borehole radar data in stratified media. Good accuracy and stability are also exhibited under sample-limited conditions, and application potential is indicated for near borehole imaging in mineral exploration, engineering geological assessment, and hydro environmental monitoring. However, the present results are still established mainly on controlled two-dimensional modeling and controlled measurement scenarios, and engineering reliability in complex field environments still needs to be verified using more realistic field BHR data. Subsequent research is focused on improving adaptability under complex geological and borehole conditions, including multilayer media, randomly rough interfaces, fractures and lenticular structures, spatially varying conductivity, and domain-randomized simulations. Transfer learning or fine tuning with a limited amount of field data is also considered in order to enhance generalization to real geological environments.

Future work is further advanced toward a physics data collaborative inversion framework. Reasonable permittivity range constraints, velocity permittivity consistency priors, Maxwell equation residual-based physics informed loss, and forward consistency checking through re-simulation of the predicted results are introduced into the network architecture and loss design in order to improve both physical credibility and field adaptability. At the same time, more systematic benchmark comparisons are conducted under a unified BHR dataset and evaluation protocol against methods such as GPRInvNet, UNet variants, and 3DInvNet. Extension toward three-dimensional borehole radar inversion is also explored through technical routes including three-dimensional CNN or three-dimensional U Net adversarial frameworks, 2.5D adjacent profile fusion strategies, and patch-wise three-dimensional training with sliding window inference. It should be noted that such an extension still faces major challenges, including the high cost of three-dimensional FDTD data generation, the substantial computational and memory burden of adversarial training, and the increased complexity of three-dimensional propagation, antenna coupling, and multiborehole spatial registration.

## Figures and Tables

**Table 1:** Quantitative metrics for circular steel target comparisons.

	Network	StratGAN	CNN	GPRNet
Metrics				
**MAE**		**2.7268**	2.9819	4.3071
$R^{2}$		**0.9431**	0.9404	0.1129
**SSIM**		0.9130	0.9090	**0.9246**
**MSSIM**		**0.9376**	0.9312	0.9073

Note: Bold values in the quantitative metrics denote the best results.

**Table 2:** Quantitative metrics for rectangular water target comparisons.

	Network	StratGAN	CNN	GPRNet
Metrics				
**MAE**		**0.7818**	0.8043	9.4435
$R^{2}$		**0.9128**	0.9033	−1.6655
**SSIM**		**0.9852**	0.9827	0.2158
**MSSIM**		**0.9948**	0.9926	0.1952

Note: Bold values in the quantitative metrics denote the best results.

**Table 3:** Quantitative metrics for triangular saturated-clay target comparisons.

	Network	StratGAN	CNN	GPRNet
Metrics				
**MAE**		0.5094	0.5396	**0.2835**
$R^{2}$		0.9460	**0.9492**	0.9146
**SSIM**		**0.9918**	0.9908	0.9851
**MSSIM**		**0.9945**	0.9941	0.9879

Note: Bold values in the quantitative metrics denote the best results.

**Table 4:** Quantitative metrics for full simulated dataset comparisons.

	Network	StratGAN	CNN	GPRNet
Metrics				
**MAE**		**1.1737**	1.1998	3.9601
$R^{2}$		**0.8784**	0.8767	−0.0038
**SSIM**		**0.9737**	0.9728	0.7152
**MSSIM**		**0.9821**	0.9815	0.7111

Note: Bold values in the quantitative metrics denote the best results.

**Table 5:** Quantitative metrics for measured rectangular steel target comparisons.

	Network	StratGAN	CNN	GPRNet
Metrics				
**MAE**		0.3293	0.3634	**0.1714**
$R^{2}$		**0.9602**	0.9586	0.4965
**SSIM**		**0.9966**	0.9956	0.9936
**MSSIM**		**0.9992**	0.9956	0.9776

Note: Bold values in the quantitative metrics denote the best results.

**Table 6:** Quantitative metrics for full measured dataset comparisons.

	Network	StratGAN	CNN	GPRNet
Metrics				
**MAE**		0.3009	0.3356	**0.1657**
$R^{2}$		**0.9533**	0.9492	0.5598
**SSIM**		**0.9962**	0.9957	0.9948
**MSSIM**		**0.9991**	0.9988	0.9856

Note: Bold values in the quantitative metrics denote the best results.

## Data Availability

Certain portions of the data are being used in ongoing related research and projects; premature disclosure may disrupt the progression of these studies or affect the outcomes of associated experiments. To ensure research integrity and orderly project advancement, the feasibility of data disclosure will be evaluated after all related research and projects are completed. For interested researchers, reasonable data requests will be assessed by the corresponding author, and the necessary data support will be provided within permissible boundaries.
